# From controversy to clinical evidence: the evolving role of cannabis in pain therapy

**DOI:** 10.1007/s00406-026-02225-x

**Published:** 2026-04-02

**Authors:** R. Demann, U. Bonnet, G. Juckel, J. Kuhn

**Affiliations:** 1 Faculty of Medicine, University of Cologne, Cologne, Germany; 2 Alexianer Hospital Cologne, Alexianer Köln GmbH, 51149 Cologne, Germany; 3 Department of Mental Health, Evangelisches Krankenhaus Castrop-Rauxel, Academic Teaching Hospital of the University of Duisburg-Essen, Castrop-Rauxel, Germany; 4 Department of Psychiatry and Psychotherapy, Faculty of Medicine, LVR-University Hospital Essen, University of Duisburg-Essen, Essen, Germany; 5 Department of Psychiatry, LWL University Hospital, Ruhr University Bochum, Bochum, Germany; 6 Department of Psychiatry and Psychotherapy, University Hospital Cologne, Kerpener Str. 62, 50924 Cologne, Germany

Cannabis has re-entered public and political debate, driving renewed interest in its therapeutic potential [1, 2], including for chronic low back pain (CLBP). CLBP is the leading cause of years lived with disability, affecting over 600 million people worldwide as of 2020 [3]. Current pharmacological treatments for CLBP are largely limited to NSAIDs and opioids, both of which are associated with substantial adverse effects [4, 5], prompting exploration of cannabis-based medicines as a potential alternative. However, evidence supporting their efficacy remains limited and heterogeneous [2, 4, 6].

Cannabis is a pharmacologically complex substance that contains various cannabinoids and other bioactive compounds acting on the endocannabinoid system and additional nociceptive signaling pathways [6]. This diversity may contribute to analgesic effects while making it difficult to attribute efficacy to specific constituents and to compare findings across studies [2].

Against this backdrop, a recently published phase 3 trial in Nature Medicine investigated VER-01, a standardized full-spectrum extract of the *C. sativa L*. strain DKJ127 (2.5 mg THC, 0.02 mg CBD, 0.1 mg cannabigerol, plus terpenes and flavonoids per dose unit), in patients with CLBP. The study’s four-phase design aimed to evaluate both short- and longer-term efficacy and safety in a comparatively large and well-characterized cohort [5].

This phase 3 trial [5] utilized a four-phase design incorporating double-blind periods, open-label extensions, and a randomized withdrawal period.

In phase A, patients were randomized in a double-blind fashion to either VER-01 or placebo for 12 weeks. Patients treated with VER-01 showed a significantly greater reduction in mean pain intensity on an 11-point numerical rating scale than those receiving placebo, therefore meeting the primary endpoint (MD = − 0.6; *P* < 0.001). The difference was more pronounced in patients with a neuropathic pain component (MD = − 1.5; *P* < 0.001), who also showed larger reductions in NPSI scores (MD = − 7.3; *P* = 0.017), and in those with severe pain (MD = − 1.0; *P* = 0.011).

This was followed by a 6-month open-label extension (phase B) in which all participants received VER-01. Thereafter, patients either entered a 6-month open-label continuation phase (phase C) or a randomized withdrawal phase (phase D). During the open-label periods, patients improved further, with those progressing to phase C maintaining this level of pain relief without dose escalation. In phase D, responders were re-randomized to continue VER-01 or switch to placebo. The primary endpoint, time to treatment failure, was not met (HR = 0.75; *P* = 0.288), likely due to limited power, although the placebo group showed a significantly greater increase in pain than the VER-01 group (MD = − 0.5; *P* = 0.034).

Outcomes in additional domains, including sleep quality, physical function, and patient global impression of change, also improved significantly in favor of VER-01 compared with placebo.

Adverse effects such as nausea and fatigue occurred significantly more often in the VER-01 group than in the placebo group (*P* < 0.001 for all). Although 94% of events were mild to moderate and became less frequent over time, discontinuations were more common with VER-01 (17.3% vs. 3.5%).

No signals of misuse, dependence, or withdrawal were observed in the VER-01 group.

This phase 3 trial of VER-01 in CLBP demonstrated a statistically significant yet small analgesic effect (MD = − 0.6; NNTB 6.8 for ≥ 30% pain reduction). Responder rates, however, compare favorably with those reported for opioids, as noted by the trial authors [5]. Importantly, VER-01 also improved physical function and sleep quality, domains in which standard pharmacological options often perform poorly. Over up to 12 months of treatment, there were no signals of dose escalation, drug abuse, dependence, or clinically relevant withdrawal, which is notable given the well-documented risk of these problems with opioids [7].

There remain several concerns regarding the use of VER-01.

Adverse events were substantially more frequent with VER-01 and contributed to a notable rate of treatment discontinuation. This relatively high rate of AE-related dropout needs to be weighed against the only small between-group effect size on pain.

The markedly higher frequency of noticeable side effects also raises concerns about functional unblinding, a possibility that cannot be evaluated, as no formal assessment of blinding was conducted. Such issues may inflate the apparent treatment effect, as shown in psilocybin studies, where unblinding was linked to disproportionately small placebo responses [8]. Expectation-driven biases could likewise have affected outcomes in the unblinded phases of this trial.

In addition, prior medical or recreational cannabis use beyond the 30-day exclusion window was not systematically characterized, leaving open the extent to which prior cannabis use may have influenced treatment response.

Importantly, the randomized withdrawal phase did not meet its primary endpoint (time to treatment failure), leaving the durability of benefit uncertain. A key methodological concern is that phase D included only responders from the preceding open-label period, which is informative from a mechanistic perspective but introduces substantial selection bias: it retains individuals who both tolerated and benefited from the drug, potentially overestimating the stability of treatment effects while underestimating longer-term harms in a broader CLBP population.

Despite the fact that head-to-head data with opioids have recently become available [9], VER-01’s place within multimodal analgesic regimens remains the subject of further investigation.

Although the chemically well-defined extract enhances internal validity, external validity remains limited because cannabis products differ widely in composition and their active constituents remain unclear. This uncertainty also hampers efforts to create more targeted formulations that might retain efficacy while reducing adverse events.

VER-01 represents a promising addition to the limited pharmacological options available for CLBP, particularly in patients with neuropathic pain features. However, its group-level benefits are moderate. These gains must be balanced against the relatively high rates of adverse events and treatment discontinuation.

Overall, this phase 3 trial represents the first well-powered study in this area (albeit only in phase A, *N* = 820) and an important step towards an evidence-based use of cannabis-derived therapeutics in medicine. Nevertheless, further studies are required to clarify the optimal clinical role of VER-01 and to address remaining questions regarding comparative effectiveness, long-term safety, and mechanisms of action.

## Data Availability

No datasets were generated or analysed during the current study.
